# Poor Muscle Status, Dietary Protein Intake, Exercise Levels, Quality of Life and Physical Function in Women with Metastatic Breast Cancer at Chemotherapy Commencement and during Follow-Up

**DOI:** 10.3390/curroncol30010054

**Published:** 2023-01-05

**Authors:** Jessica Parkinson, Amelia Bandera, Megan Crichton, Catherine Shannon, Natasha Woodward, Adam Hodgkinson, Luke Millar, Laisa Teleni, Barbara S. van der Meij

**Affiliations:** 1 Bond University Nutrition and Dietetics Research Group, Faculty of Health Sciences and Medicine, Bond University, Gold Coast, QLD 4226, Australia; 2 Mater Health, Brisbane, QLD 4101, Australia; 3 Mater Research Institute, University of Queensland, Brisbane, QLD 4072, Australia; 4 Department of Nutrition, Dietetics and Lifestyle, HAN University of Applied Sciences, 6525 EN Nijmegen, The Netherlands; 5 Division of Human Nutrition and Health, Wageningen University and Research, 6708 PD Wageningen, The Netherlands

**Keywords:** malnutrition, sarcopenia, metastatic breast cancer, physical function, quality of life

## Abstract

This study aimed to investigate nutritional status, body composition, dietary protein intake, handgrip strength, 6 min or 4 m walk tests, self-reported physical activity, physical function, and quality of life (QoL-EORTC-QLQc30) at commencement of chemotherapy; to detect changes over time (from commencement of chemotherapy, and after 3, 6, 12, 26 and 52 weeks) in women with metastatic breast cancer (MBC); and to investigate the relationship between nutritional variables. ‘Sarcopenia’ was defined as low muscle mass and strength, ‘myosteatosis’ as muscle fat-infiltration (CT scan). Continuous variables were analysed using paired t-tests between baseline and follow-ups. Fifteen women (54y, 95% CI [46.3;61.2]) were recruited. At baseline, malnutrition was present in 3 (20%) participants, sarcopenia in 3 (20%) and myosteatosis in 7 (54%). Thirteen (87%) participants had low protein intake; low handgrip strength was observed in 0, and low walk test distance and physical activity in four (27%) participants. Physical function and QoL were low in 10 (67%) and 9 (60%), respectively. QoL between baseline and 52 weeks decreased by 11.7 (95% CI [2.4;20.9], *p* = 0.025). Other variables did not significantly change over time. In this small study sample, myosteatosis, low dietary protein intake, low exercise levels and impaired quality of life and physical function are common.

## 1. Introduction

In Western countries, breast cancer is the most common form of cancer among women, and almost 30% of those diagnosed will progress to metastatic disease [1,2]. Weight gain is common in women with breast cancer, and a higher body weight is associated with decreased quality of life (QoL), cancer recurrence, and shorter survival [3,4]. There is evidence that overweight and obesity predict poor outcomes in women with early stage disease, linked to the production of pro-inflammatory cytokines, growth factors and hormones influencing the tumor microenvironment and inducing cancer progression. In postmenopausal women, estrogen produced by adipose tissue is related to the occurrence and progression of breast cancer [5]. However, the same has not been seen in women with metastatic breast cancere, in which case there may be a protective effect of a higher body weight [1]. Conflicting data have been found regarding the prognostic impact of overweight or obesity in metastatic disease. Results of a study of 82 women with metastatic breast cancer (MBC) in 2017 found that being overweight improved overall survival when compared to women with a BMI ≤ 25kg/m^2^ [6]. However, recent work by Saleh et al., the largest assessment conducted exploring impacts of BMI on MBC survival outcomes in 12,999 women with BC, showed that overweight and obesity were not associated with poorer outcomes, while patients with underweight had a shorter overall and progression-free survival [7]. The possible protective nature of overweight and obesity in patients with MBC requires further exploration. Sarcopenia, however, has been shown to have a high prevalence and predict negative outcomes in all cancers, including breast cancer [8,9,10]. Sarcopenia is defined as an acute or chronic muscle disease, characterised by low muscle strength [11]. Excessive loss of muscle mass among cancer patients is common [12]. In advanced cancer as a whole, sarcopenia prevalence is 19–74% and skeletal muscle mass has been found to be lower in metastatic breast cancer (MBC) compared to early stage breast cancer [1,13]. Studies in women with breast cancer have found that the loss of skeletal muscle mass is a predictor of poor outcomes [13,14]. Sarcopenia in cancer patients is associated with increased risk of toxicity during treatment, physical disability, extended hospitalisation, lower QoL, cancer progression, and reduced survival [12,13,15,16].

Loss of muscle mass can be caused by many factors including aging, hormonal changes, altered energy expenditure, low dietary intake, disease-related inflammation, treatment toxicities, and lack of physical activity [16]. Low muscle mass is often accompanied by reduced physical function, physical exercise and muscle weakness [17].

Physical exercise is essential for muscle maintenance. Studies have shown physical activity reduces when chemotherapy commences, even though exercise is safe in patients undergoing chemotherapy and the benefits of exercise for cancer patients are well known [18,19]. Physical activity can improve fatigue, bone health, muscle strength, and QoL in patients with breast cancer [20,21,22,23]. The European Society for Clinical Nutrition and Metabolism (ESPEN), the American Cancer Society, and the Breast Cancer Network Australia recommend maintaining or increasing physical activity and aiming for at least 150 min of moderate intensity exercise per week [17,24]. Some studies have shown that patients with breast cancer lack willingness to participate in exercise, creating a barrier to exercise interventions [17]. There could also be a relationship between QoL and physical activity, with low perceived QoL reducing willingness to exercise, and low exercise frequency reducing QoL [23]. However, a recent trial in patients with MBC showed a high adherence, improved walking distance and muscle strength and reduced BMI and hip circumference [25].

As well as experiencing issues with QoL and physical exercise, women with breast cancer are also at risk of malnutrition. Malnutrition in cancer patients can occur as a result of increased protein and energy requirements and/or decreased intake, either as a result of the tumour itself and/or the side-effects of treatment [26,27]. Malnutrition is characterised by weight loss and muscle wasting [26]. Malnutrition is associated with lower response to treatment, higher toxicity, poorer survival, and poorer QoL [26,27,28]. In a study by Hebuterne et al. malnutrition was found to be present in 20.5% of breast cancer patients. In the Hebuterne et al. [26] study, breast cancer patients had one of the lowest rates of malnutrition, however, only 44% of participants had metastatic disease. A study by Harvie et al. [29] in MBC patients found that meeting or exceeding energy requirements led to gains in body fat, however, did not prevent the loss of muscle mass. This group showed no change in overall weight, while gaining fat and losing muscle mass [29]. Knowing this, and that preservation of muscle mass is associated with improved functional capacity and QoL, further demonstrates the importance of nutrition and exercise for muscle maintenance in breast cancer patients [29].

As compared to early stage breast cancer, few analyses of body composition changes have been conducted in the metastatic setting, therefore little is known about the impact of MBC on body composition and the relation with other patient-centered outcomes [1]. A few studies have looked at the effects of chemotherapy on body composition in MBC. One study found that muscle attenuation decreased, indicating fat infiltration in the muscle. In this study, low muscle mass did not show significant associations with overall survival whereas low muscle attenuation did [30]. Another study found a statistically significant gradual decline in muscle mass over a maximum period of 1500 days. The average decline in muscle mass was 5.0 ± 2.5 cm^2^ per year, which suggests a loss much greater than aging-associated muscle mass loss [31]. Relatively few studies have investigated the prognostic role of muscle mass in women with MBC [1,13,30]. Meta-analyses of 3 studies showed MBC patients with sarcopenia had more chemotherapy toxicity than non-sarcopenic patients, and time to tumour progression was nearly 71 days longer in non-sarcopenic patients [32]. Because little is known about body composition changes, health professionals are unable to provide evidence-based care, which reduces the likelihood of improved outcomes for these patients. Therefore, the aims of this study were:

(1) To investigate nutritional status, body composition, physical function, and quality of life at commencement of chemotherapy and to detect changes over time in MBC patients;

(2) To investigate the relationship between body composition, physical function, nutritional status, and quality of life.

## 2. Materials and Methods

### 2.1. Setting

This prospective observational cohort study was reported according to the STROBE guidelines [33]. It was carried out at the Mater Cancer Care Centre in Brisbane, Queensland (Australia) between October 2017 and March 2020. Women undergoing chemotherapy for MBC were recruited and completed their first round of assessments upon commencement of a new chemotherapy regimen and assessed again at 3, 6, 12, 26, and 52 weeks.

### 2.2. Participants

The following inclusion criteria were applied for participants to be eligible for inclusion in this study; female, above 18 years of age, metastatic (stage IV) MBC (primary), life expectancy greater than 3 months, mobile, Eastern Cooperative Oncology Group performance status of 0, 1, or 2 (confirmed by the participant’s oncologist), commencing a new chemotherapy regimen where a minimum 2 cycles of chemotherapy were scheduled, and able to speak, read and write English. Participants were excluded if they had a permanent pacemaker or other medical implant not appropriate for bio-impedance spectroscopy (BIS) assessment, severe cognitive or intellectual disability or mental illness, presence of acute illness or unstable chronic illness, or any other condition that would interfere with the study or safety of the patient according to the principal investigator, nurse, or patient.

### 2.3. Data Collection

General characteristics including age, height, weight, BMI, and ethnicity were collected at baseline. Height was collected using a stadiometer to the nearest 0.5 cm with the patient having removed their shoes and standing with their arms hanging by their sides and their back to the stadiometer. The participant had their feet slightly apart with back of heels, middle of shoulders, buttocks and back of head touching the stadiometer, and facing straight ahead with neck straight and head in the Frankfort plane. Participants’ weight was collected using a digital scale (Wedderburn WM303H, Willawong, QLD, Australia) and recorded in kg to the closest one decimal. Baseline BMI was calculated by dividing weight (kg) by squared height (m^2^). Other baseline information (stage of disease, treatment regimen, and medical history) was collected from the participant’s medical records.

### 2.4. Nutritional Status

Malnutrition was determined using the Malnutrition Screening Tool (MST) [34] and the Patient-Generated Subjective Global Assessment (PG-SGA) [35] at each time point. These questionnaires were completed either by a dietitian or student dietitian through an interview with the participant. The MST gives a numerical score between zero and four to determine a person’s risk of malnutrition, with a score of >2 equating to ‘at risk of malnutrition’. The PG-SGA provides both a numerical score to assist in triaging and monitoring changes in nutritional status and the categories A, B, and C, to diagnose as well-nourished, mild-moderate malnutrition, and severe malnutrition, respectively. The numerical score ranges from 0–55, with a higher score indicating more serious malnutrition signs and symptoms.

Dietary protein and energy intake was determined using the five-pass dietary recall methodology to record a “typical” day of dietary intake of one weekday and weekend day. Energy and protein intake was assessed using the Xyris software in Foodworks™ or Easy Diet Diary™ programs. Dietary energy and protein intake analysed in the Xyris software was compared to the recommended protein and energy requirements based on NEMO guidelines of ≥125 kJ/kg (adjusted) body weight, and ≥1.2 g protein/kg (adjusted) body weight for adults undergoing chemotherapy [36].

### 2.5. Body Composition

Body composition was assessed using bio-impedance spectroscopy (BIS) and computed tomography (CT) scanning. BIS was completed at each time point, and CT scans were performed as part of usual care. BIS was completed using tetra polar (ankle-foot) multiple frequency BIS device (Impedimed SFB7, Carlsbad, California, USA) with the patient in a supine position, following a five-minute period of laying down. The participants were instructed to remove all metal, jewellery, and shoes, and to empty their bladder prior to the assessment. A user manual was developed by the research team to be followed to reduce measurement bias (Supplementary Material S1). Fat-free mass (FFM) in kgs, fat mass (FM) in %, extracellular water (ECW) and intracellular water (ICW) in litres were read from the Impedimed SFB7 device.

The results of the CT scans were obtained for the date closest to the participant’s study follow-ups. Analysis of the CT images was performed using SliceOmatic (TomoVision, Magog, QC, Canada) with a single axial abdominal CT image landmarked at L3. A trained observer reviewed and analysed all the images and a second observer double checked the accuracy of the analyses. Both observers were trained and blinded to patient outcomes. Interrater coefficients of variation were within the expected ranges (Skeletal Muscle Index, SMI: 2%; Skeletal Muscle Ratio, SMR: 4%; Visceral Adipose Tissue Index, VATI: 4–7%) [23,24]. The predetermined Hounsfield Unit (HU) thresholds were −29 HU to +150 HU for skeletal muscle (SM), −150 to −50 HU for visceral adipose tissue (VAT), and −190 to −30 HU for subcutaneous adipose tissue (SAT) and intermuscular adipose tissue (IMAT) [37,38]. Findings for SM, VAT, SAT and IMAT area were reported in cm^2^ and mean skeletal muscle radiodensity (SMR) was reported in HU.

### 2.6. Physical Function Tests

A hand grip dynamometer (JAMAR plus+, JAMAR, Hatfield, USA) was used to measure peripheral muscle strength. A user manual was developed for investigators in order to reduce measurement bias (Supplementary Material S2). Participants were asked to use their non-dominant hand, and whether this occurred was recorded on the case report form. The measurement followed a standard procedure, with the participant’s arm at a 90-degree angle and with standardised encouragement being provided by the investigator. The test was repeated three times, and the result of each attempt was recorded. Handgrip strength was used as a component in the diagnosis of sarcopenia in patients; the highest grip strength value was compared with the cut-off for sarcopenia from the revised European Working Group on Sarcopenia in Older People (EWGSOP2) guidelines (Supplementary Material S3); i.e., a handgrip strength <16 kg indicates in females indicates a low muscle strength and potentially sarcopenia [11].

Physical function was assessed using either the six-minute walk test (6MWT) or a four m walk test (gait speed); the latter being used in the event that the participant refused the 6MWT or was not able to perform the 6MWT. Blood pressure was measured prior to commencing the 6MWT to ensure the participant was fit to participate. Oxygen saturation and heart rate were measured using a ChoiceMMed sats monitor (ChoiceMMed Germany, Düsseldorf, Germany) and perceived exertion was measured using the revised category-ratio Borg scale every minute during the walk test [39]. Based on previous studies in cancer patients and healthy adults by Jones et al. [40] and Kaysmjanova et al. [41], a cut-off of <400 m was used for the 6MWT as it has been shown to be a possible prognostic factor for survival. The cut-off value used for the four m walk test was ≤0.8 m/s as per the EWGSOP2 revised guidelines (Supplementary Material S3) [11]. Three categories were then created: did not participate (refused/unable), above 400 m or 0.8 m/s, and <400 m or <0.8 m/s.

Self-reported physical activity levels were assessed using the Godin-Shephard Leisure-Time Physical Activity Questionnaire, of which the questions were asked to the participant verbally by a researcher. Leisure Activity Scores (LAS) were calculated based on the participant’s self-reported mild, moderate, and strenuous exercise over the previous week: (frequency of mild × 3) + (frequency of moderate × 5) + (frequency of strenuous v 9). Scores > 24 indicated a participant was ‘active’, scores ≤ 23 indicated a participant was ‘insufficiently active’ [42,43].

### 2.7. Definition of Sarcopenia and Myosteatosis

Sarcopenia was defined using both the EWGSOP2 guidelines (Supplementary Material S1) and the third lumbar vertebra (L3) skeletal muscle index sex-specific cut-off proposed by Prado et al., which is ≤38.5 cm^2^/m^2^. This cut off has previously been used in the oncology setting and associated with mortality [8]. The EWGSOP2 guidelines define sarcopenia as low muscle strength (for which we applied handgrip strength) and low muscle mass (as indicated by skeletal muscle index).

Myosteatosis was defined as (1) skeletal muscle radiodensity < 41 HU in subjects with BMI < 25 kg/m^2^ and <33 HU for subjects with BMI ≥ 25 kg/m^2^ [44] and (2) IMAT ≤ 3.5 cm^2^/m^2^ [45].

### 2.8. Quality of Life

QoL was assessed at each timepoint, as the participant’s very first assessment on the first day of data collection, to reduce the risk of other assessments, appointments, or treatments influencing the results. Participants completed the self-administered EORTC QLQ-C30 questionnaire, which is both cancer-specific and validated in the oncology population [46]. The EORTC QLQ-C30 questionnaire includes functioning scales, and a global QoL assessment. These data were then entered into a Microsoft Excel spreadsheet and scores were calculated according to the EORTC QLQ-C30 manual [47]. Only the global QoL score and physical function score were used for this study. The global QoL score represents the participant’s view of their own health and QoL, and the physical function score represents the participants’ ability to complete activities of daily living. A higher score represents a better QoL and higher level of functioning, with the possibility to score between 0 and 100. Scores were compared with reference ranges for women with MBC (mean (SD)): 60.2 (25.5) for QoLand 81.6 (18.7) for physical functioning) [47].

### 2.9. Statistics 

This study was convenience sampled. The sample size was calculated using the estimated range of sarcopenia prevalence in MBC of 25% [40] to 58% [44]. The average of 40% was derived from this range, and using an estimated dropout rate of 20%, the final target sample size to recruit for this study is 45. From this number of participants, it was expected that 15 would be identified to have sarcopenia.

The software SPSS statistics version 26 was used for data analysis (IBM, Chicago, Ill, USA). First, data was tested for normality using Shapiro–Wilkinson tests. Normally distributed continuous variables were described as Mean (SD), and non-normal continuous variables were described as Median (IQR) or Median (range). Categorical variables were described as n (%). Paired *t*-tests for continuous and Chi-square tests for categorical variables were completed between baseline and follow up time points. For data that was not normally distributed, the Wilcoxon signed rank test was used to compare average values between time points. Associations between all variables were tested through Pearson’s bivariate correlation analysis. All data was included in the analysis, including that of participants who withdrew at later stages of the study.

To allow for percentage change calculations to be run, when a participant had an activity score of zero at baseline, it was changed to one as it is not possible to divide by 0.

### 2.10. Ethics Statement 

This study was approved by the Mater Misericordiae Limited Human Research Ethics Committee (REF: HREC/17/MHS/63) and has been performed in accordance with the ethical standards as laid down in the 1964 Declaration of Helsinki and its later amendments.

Eligible patients were asked for permission to be contacted about the study and if so, were referred to the research team by the clinical oncologists (CS or NW). A member of the research team then provided verbal and written information about the study purposes and processes in lay terms. If after consideration the patient agreed to participate, a follow up visit/baseline assessment was scheduled to sign the informed consent form and perform baseline assessments.

## 3. Results

**Figure 1:**
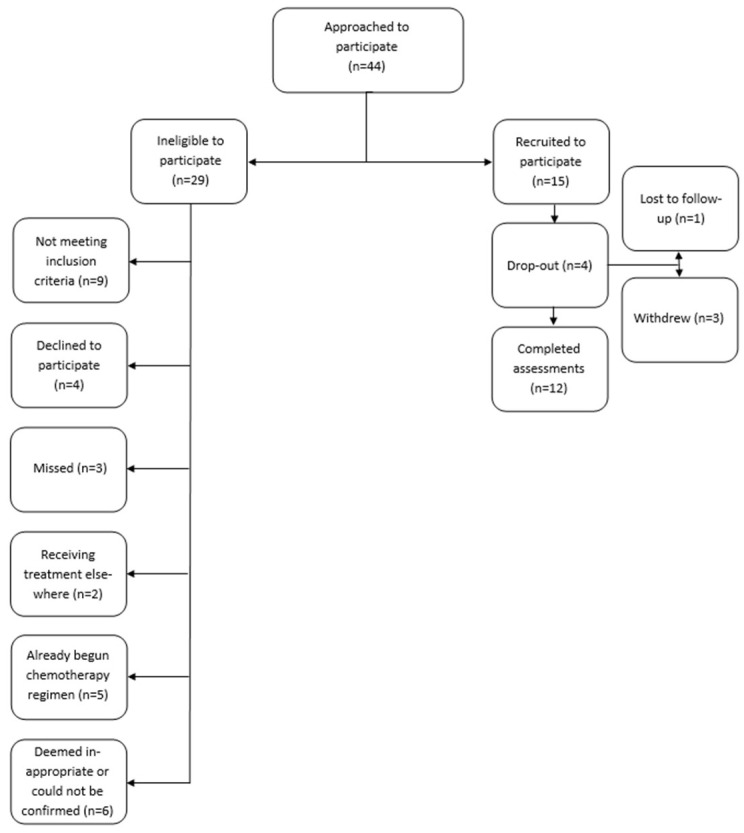
Flow chart.

Within the 21-month recruitment period between November 2017 and August 2019, 44 women were approached to participate. Of these 44, 15 were enrolled, with the reasons for not participating being: refused (*n* = 4), missed (*n* = 3), deemed inappropriate or could not be confirmed to be appropriate (*n* = 6), already started a new chemotherapy regimen (*n* = 5), receiving their treatment elsewhere (*n* = 2), and not meeting other inclusion criteria (9) (Figure 1). Baseline characteristics of participants are outlined in Table 1. The mean age was 54 years (range 33–76) and the mean (SD) BMI was 29.8 kg/m^2^ (7.4). As shown in Figure 1, 4 participants of the 15 recruited dropped out; with 3 participants withdrawing and 1 participant being lost to follow up.

### 3.1. Nutritional status

**Figure 2:**
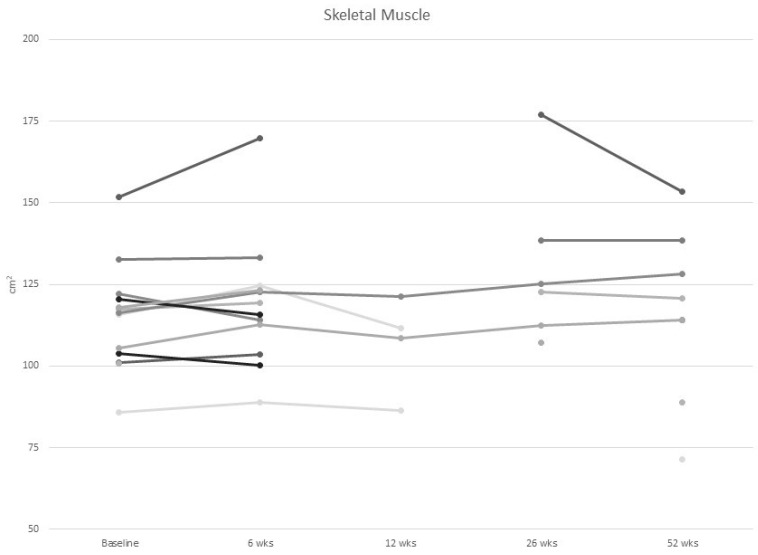
Individual values of skeletal muscle mass in women with metastatic breast cancer from baseline to 52 wks of follow up.

**Figure 3:**
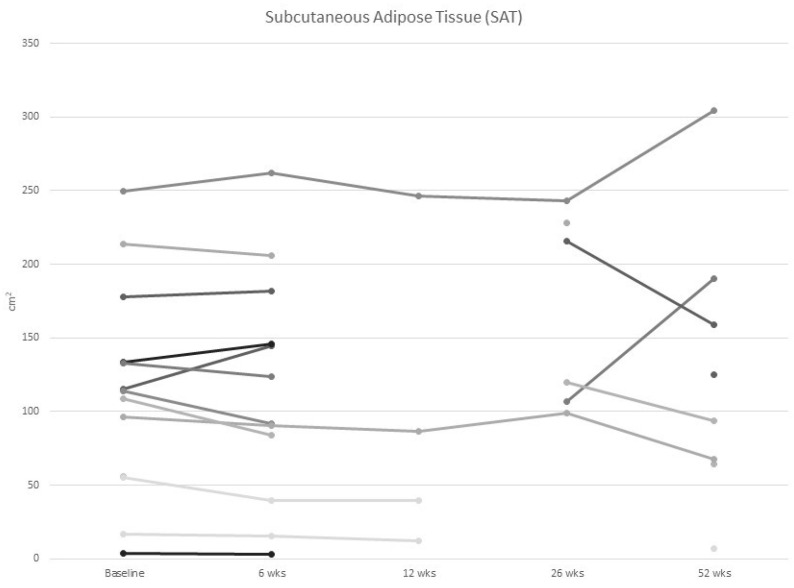
Individual values of subcutaneous adipose tissue in women with metastatic breast cancer from baseline to 52 wks of follow up.

**Figure 4:**
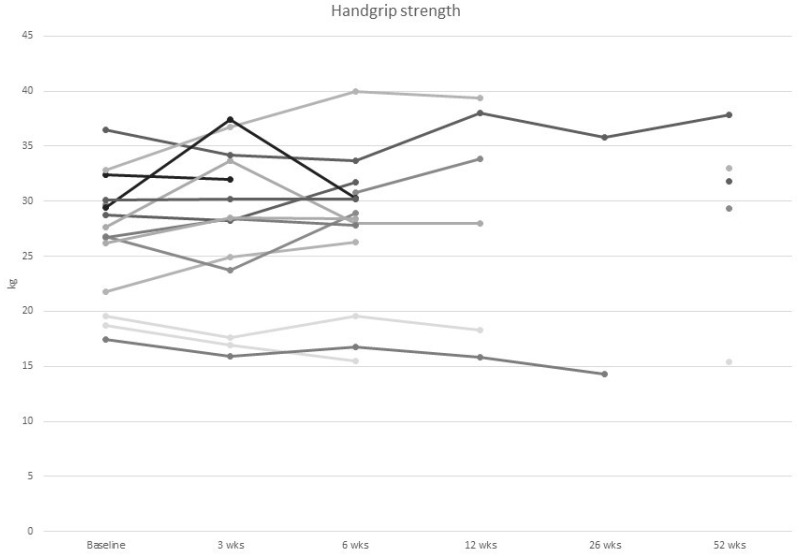
Individual values of handgrip strength in women with metastatic breast cancer from baseline to 52 wks of follow up.

**Figure 5:**
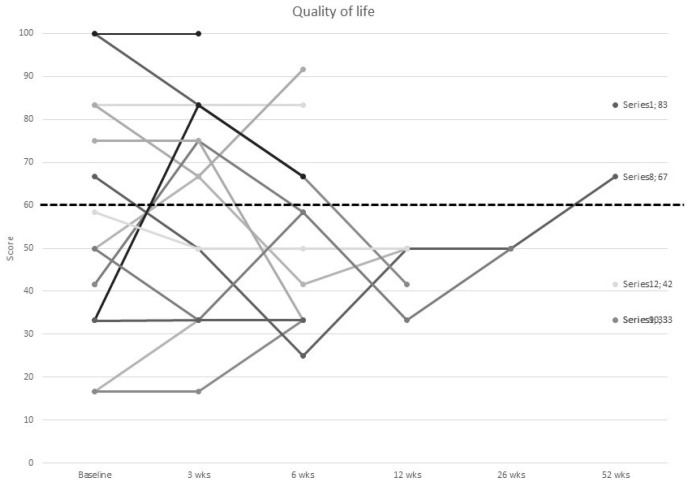
Individual values of quality of life in women with metastatic breast cancer from baseline to 52 wks of follow up.

At baseline, one-third of participants were at risk of malnutrition and 20% were moderately malnourished. None of the participants were severely malnourished. There was no change in body weight nor in the observed prevalence of mild or moderate malnutrition over the 1-year study period. Participants at risk of malnutrition at baseline did not show a difference in weight, muscle mass, muscle strength or quality of life during follow-up (Figure 2, Figure 3, Figure 4 and Figure 5).

Dietary recalls indicated that participants met 58% of their energy requirements at baseline (Table 2), 43% at 12 weeks (about 3 months), and 78% at 12 months (Table 3). For dietary protein, participants met 58% of their protein requirements at baseline, 52% at 12 weeks, and 79% at 12 monthtable. Correlation testing found that fat free mass and physical activity were negatively correlated at baseline (*r* = −0.65, *p* = 0.008), and skeletal muscle and physical function were negatively correlated at six weeks (*r* = −0.70 *p* = 0.01).

### 3.2. Body Composition

As shown in Table 3, sarcopenia based on the EWGSOP2 definition using BIS fat free mass index was present in 1 (6.6%) of the 15 participants at baseline and in zero of the five participants at one year. Using the Prado definition using skeletal muscle index from CT scans, three (20%) participants had sarcopenia at baseline and two (25%) had sarcopenia at 12 months (Table 3. Based on the amount of IMAT, the majority (73%) of women had myosteatosis at baseline. BIS results showed that fat free mass increased by 4.1% (14.6, *p* = 0.60), and fat mass decreased by 4.4% (28.2, *p* = 0.79) between baseline and 12 months. The CT scan results showed skeletal muscle increased by 1.4% (10.5, *p* = 0.56) (Figure 2) and visceral adipose tissue decreased by 3.0% (32.3, *p* = 0.55) over 12 months (Figure 3).

### 3.3. Physical Function Tests

Hand grip strength at baseline was above the age-specific reference value in all participants and it did not significantly change over time (+1.56% (12.0) over the year, *p* = 1.00) (Figure 4). Four out of 15 participants (26%) at baseline and three out of five participants (60%) at 1 year either refused the walk tests or were below the cut-off of at least 400 m (Table 3). The average distance for the 6MWT was comparable between baseline and 1 year (+1.44% (18.4), *p* = 0.99). Gait speed was not used to diagnose severe sarcopenia due to the small number of participants who completed the walk test (<5 participants at each time point). Only 4 of the 15 participants (27%) met, or exceeded the cut-off for physical activity at baseline, meaning 73% of participants were not exercising enough. This increased to 60% at 1 year, when 3 out of 5 were above the cut-off. Twenty percent of participants reported that they were completing minimal physical activity at baseline, increasing to 40% at 12 months. Reported physical activity (LAS) increased by 32.0 (43.9) points between baseline and 1 year (*p* = 0.18).

### 3.4. Quality of Life

Nine (60%) out of 15 participants had QoL scores below the reference values representative of a general population of MBC patients at baseline (Table 3). After 12 months, QoL score significantly decreased by 11.7% (SD, *p* = 0.03), with three (60%) out of the remaining five participants reporting scores below the reference range (Figure 5). Physical function results from the EORTC QLQ-C30 questionnaire showed that ten (67%) out of 15 participants were below the physical function score cut-off for MBC patients at baseline (Table 3). At 12 months, four (80%) participants were below the cut-off for physical function. 

### 3.5. Prognostic Impact of Malnutrition Risk at Baseline

Participants at risk of malnutrition at baseline did not show a difference in body weight, muscle mass, muscle strength, or quality of life after 52 weeks, compared to those not at risk. Similarly, participants with malnutrition, low muscle mass or muscle strength at baseline did not have a higher quality of life reduction than participants who did not have malnutrition, low muscle mass or muscle strength at baseline. Due to the small sample size (only 5 observations after 52 weeks), the power was too low to detect significant differences.

## 4. Discussion

This study found a low prevalence of malnutrition and sarcopenia and a poor quality of life at the start of a new chemotherapy regimen in a small group of women with MBC. After 1 year of treatment, the majority of women had not lost weight, had no sarcopenia, and were well-nourished. However, most participants’ activity levels, physical function, protein and energy intake, and QoL scores were below the reference range at baseline and over time. These findings indicate long-term suboptimal physical function, quality of life and nutritional intake in this small sample of women with MBC and correspond to those of the few similar studies that have been performed in this population [23,48]. In a longitudinal mixed methods study, de Kruijf et al. [49] documented impaired food intake and fatigue, and issues with taste, smell, and appetite in some, but not all women with breast cancer undergoing surgery and chemotherapy. In contrast with our study, 40% had low physical activity levels [49].

Body composition is a hallmark of nutritional status and has a prognostic value in patients with cancer [50]. We used two techniques to assess body composition in this study, BIS and CT scans. Sarcopenia results differed by 2–55% between the CT scans and the BIS however both the CT scans and BIS results showed less than a 5% change in fat free mass (FFM) over time. The Prado definition of sarcopenia using SMI is often used in oncology populations, however as the timing of CT scans was not always aligned with the set timepoints, the BIS assessments and EWGSOP2 guidelines were also used to diagnose sarcopenia. The Prado cut off for sarcopenia identified more participants as sarcopenic compared to the EWGSOP2 guidelines. CT scans are considered the gold standard in muscle assessment [11,38,51] and using the Prado cut-off for sarcopenia, 17–75% of participants were identified as having sarcopenia at any time point in this study. These results differ from those using the BIS assessment and EWGSOP2 guidelines, which identified 0–50% as having sarcopenia. In other studies in patients with breast cancer, sarcopenia prevalence was up to 48.8% in early stage breast cancer, and up to 58% in MBC [1,13]. In other small studies in women with MBC, sarcopenia was present in 32% [23] and 34% [48] and myosteatosis in 49% [48]. In our study, 40 to 100% of the patients in our study had myosteatosis, which is alarming, as myosteatosis is even more strongly correlated to shorter survival than sarcopenia [32].

Rates of malnutrition found in this study were relatively low, between 0 and 20% at baseline or follow-up visits. Previous studies in breast cancer, mostly conducted in first world countries, report malnutrition rates that range from 19.2% to 63.5% with the higher rates being in patients that have metastatic disease and are undergoing treatment [52,53]. However, a previous study on the nutrition status of MBC found that most participants were well-nourished (92%), which is in line with the findings in this study [23]. Further studies are needed in this area with large sample sizes to be able to confidently predict changes in nutrition status in MBC patients. The mean BMI of participants at baseline was in the ‘overweight’ and ‘obese’ categories, which is typical for women with MBC [6,8,29]. Previous studies have shown that carrying extra weight may have a protective effect for MBC patients [1,6].

Participants’ reported energy and protein intakes were below requirements at all timepoints, ranging from 43% to 79%. This could be the result of issues with the taste and structure of food, as was reported by women with early stage breast cancer in a study by De Kruijf et al. [49]. This study also highlighted the relationship between poor pre-treatment nutrition knowledge or intake and low energy intake during treatment [49]. This highlights the need for more nutrition education in this population, which is supported by a qualitative study in MBC in which participants reported nutritional problems and uncertainty on how to cope with these [54]. The percentage intake of requirements of energy and protein were highest at 1 year. This could be explained by enrolling participants at the start of a new chemotherapy regimen, which could have been a time of disease progression leading to the loss of the sickest participants and the stabilisation of disease in the remaining participants.

Despite most participants having good nutrition and muscle status, their physical function as indicated by walk tests and QoL questionnaires was low. Gait speed was used as an alternative to allow for an alternative physical function test for participants who were not able to complete the 6MWT. The walk test results showed that the majority of participants were above the cut-off at baseline, however this worsened over time. The physical function score from the EORTC QLQ-C30 questionnaire showed that the ability to complete normal daily activities was low. This can have an impact on physical activity levels, which in turn, leads to muscle wasting [51], which as previously mentioned, can impact on QoL in patients with cancer. On the contrary, we found a significant negative correlation between muscle mass and both physical activity at baseline and physical function at the six-week time point.

Reported QoL was poor. QoL decreased over the year, and when compared to the MBC-specific reference ranges, was low for 60% of participants at baseline and 1 year, and up to 100% at 12 weeks. This study supports previous observations that women with incurable breast cancer experience deteriorations in QoL [55], and despite the improved treatments and supportive care, ongoing research is needed to further improve outcomes.

Studies have shown that physical activity is a strong predictor of survival in cancer patients [40,56,57,58]. This highlights the importance of breast cancer patients, especially those with metastatic disease, completing sufficient physical activity. In line with a previous study [23], physical activity levels in this population were exceptionally low for 73% of participants at baseline and 60% at 1 year. The number of participants reporting no exercise at all decreased from three to two between baseline and one year with an average 32-point increase in reported physical activity levels over the year. Low willingness to participate could be a barrier to implementing exercise interventions [20]. However, barriers and enablers for physical exercise were not explored in this study. Because we do not know what the participants’ usual exercise levels were before developing cancer, it is unclear whether the reported low exercise levels are related to the disease.

The changes that were observed in this study were not as hypothesised. Most of the changes were minor and were not statistically significant. One probable reason for this is that the women who agreed to partake in the study had less progressive disease and therefore less muscle wastage than women who declined to participate, which could have led to selection bias.

This study had several limitations. The first is that it was not possible to complete CT scans on the exact date specified for assessments, meaning that the sarcopenia diagnosis from CT results may not accurately represent the time points. The other limitation of this study is the small sample size of 15, which was lower than expected over a two-year recruitment period. It is therefore impossible to translate results to the wider population of women with MBC, and it is underpowered to detect changes. One of the reasons for this small sample size and low rate of recruitment was that patients were required to have just commenced a new chemotherapy regimen, and candidates were often missed because they were not referred on time. In addition, this study was not funded so staff availability was too low to recruit on an ongoing basis. Both pre- and post-menopausal women were recruited in order to expand recruitment opportunity. Unfortunately, menstruation status was not recorded for patients included in the study. As menopause is associated with a decline in estrogen levels which may contribute to sarcopenia, future studies considering menstruation cycles should be explored.

## 5. Conclusions

There is currently little research on changes in nutritional status, physical function, and physical characteristics in MBC patients. Therefore, the information required to optimise treatment strategies for nutrition and exercise for MBC is limited. In order to understand these changes and inform treatment strategies, this study assessed changes in body composition, muscle strength, physical function and physical activity levels, and other related patient-centred outcomes of malnutrition and QoL in MBC patients undergoing chemotherapy. After 1 year of treatment, most participants did not lose weight, did not have sarcopenia, were well-nourished, and their overall QoL improved compared to their first chemotherapy cycle. However, most participants’ activity levels, physical function, and QoL scores were below the reference ranges at baseline and over time.

## Figures and Tables

**Table 1:** General characteristics of metastatic breast cancer patients undergoing chemotherapy.

Characteristic ^1^	*n* = 15
Age (y)	53.73 (13.5) Range 33–76
Height (m)	1.62 (0.07)
Body Weight (kg)	78.3 (18.2)
BMI (kg/m^2^)	29.8 (7.4)
BMI categories, *n* (%)	
Underweight	0 (0)
Normal weight	5 (33.3)
Overweight	3 (20.0)
Obese	7 (46.7)
Race, *n* (%)	
Caucasian	11 (73.3)
Asian	2 (13.3)
Pacific Islander	2 (13.3)
Treatment pathway, *n* (%)	
Oral	1 (6.7)
Intravenous	14 (93.3)
Chemotherapy agents	
Taxane	4
Anthracycline	2
Capecitabine	2
Trastuzumab	3
Carboplatin gemcitabine	1
Eribulin mesylate	2
Nutritional Supplements ^2^, *n* (%)	
Yes	6 (40.0)
No	9 (60.0)
Steroids, *n* (%)	
High dose	0 (0.0)
Low dose	2 (13.3)

^1^ Results are mean (SD) or *n* (%). ^2^ Nutritional Supplements: A range of supplements including calcium, B vitamins, selenium, fish oil, vitamin D, and/or magnesium.

**Table 2:** Dietary intake, body composition, muscle strength, physical function and quality of life in participants with metastatic breast cancer at baseline.

Mean (SD) or *n* (%)	*n* = 15
Dietary Intake	
Energy (% of requirements) mean (SD)	57.9 (22.6)
Protein (% of requirements) mean (SD)	58.2 (20.3)
Body Composition	
Bioimpedance Spectroscopy	
Fat free mass (kg) mean (SD)	52.4 (8.4)
Fat free mass index (kg/m^2^) mean (SD)	20.0 (3.6)
Fat mass (%) mean (SD)	31.9 (7.2)
ICF (I) mean (SD)	22.1 (3.8)
ECF (I) mean (SD)	16.3 (2.5)
CT-scans	
Skeletal muscle (cm^2^) mean (SD)	114.7 (16.4)
Sarcopenia *n* (%)	3 (20.0)
IMAT (cm^2^) mean (SD)	6.6 (4.0)
SAT (cm^2^) mean (SD)	272.2 (108.5)
VAT (cm^2^) mean (SD)	113.3 (71.8)
Radiodensity—skeletal muscle (HU) mean (SD)	35.3 (7.0)
Muscle strength	
Handgrip strength (kg)	27.0 (5.5)
Physical function	
6 min walk test (m)	480.2 (86.0)
6 min walk test below cut off *n* (%)	4 (26.7)
LAS median (range)	6 (0–76)
Quality of life	
Global quality of life	56.7 (27.3)
Physical function	74.2 (19.7)

ICF, intra-cellular fluid; ECF, extracellular fluid; IMAT, intermuscular adipose tissue; PG-SGA, Patient-Generated Subjective Global Assessment; SAT, Subcutaneous adipose Tissue; VAT, visceral adipose tissue, LAS, Leisure Activity Score.

**Table 3:** Assessment of changes in nutritional status, dietary intake, body composition, physical function and quality of life in participants with metastatic breast cancer during chemotherapy.

*n* (%)	Baseline *n* = 15	3 wks *n* = 14	6 wks *n* = 14	12 wks *n* = 5	26 wks *n* = 2	52 wks *n* = 5
Nutritional status						
Malnutrition screening (MST)						
At risk (%)	5 (33.3)	2 (14.3)	5 (35.7)	0 (0.0)	0 (0.0)	0 (0.0)
Malnutrition assessment (PG-SGA)						
A—well nourished	12 (80.0)	12 (85.7)	11 (78.6)	5 (100.0)	n/a	4 (80.0)
B—moderately malnourished	3 (20.0)	2 (14.3)	3 (21.4)	0 (0.0)	n/a	1 (20.0)
C—severely malnourished	0 (0.0)	0 (0.0)	0 (0.0)	0 (0.0)	n/a	0 (0.0)
Dietary intake						
Met energy requirements	2 (13.3)	3 (21.4)	2 (14.3)	0 (0.0)	0 (0.0)	1 (20.0)
Met protein requirements	2 (13.3)	2 (14.3)	3 (21.4)	0 (0.0)	0 (0.0)	1 (20.0)
Body Composition						
IMAT above cut-off (myosteatosis)	11 (73.3)	n/a	10 (71.4)	4 (80.0)	2 (100.0)	5 (100.0)
Skeletal muscle radiodensity below cut-off (myosteatosis)	7 (46.7%)	n/a	7 (50.0)	2 (40.0)	2 (100.0)	4 (50.0)
Sarcopenia (EWGSOP-criteria)	1 (6.7)	1 (7.1)	2 (14.3)	1 (20.0)	1 (50.0)	0 (0.0)
Sarcopenia (CT-scans)	3 (20.0)	n/a	2 (14.3)	3 (60.0)	0 (0.0)	2 (40.0)
Physical function						
Handgrip strength below cut-off	0 (0.0)	1 (7.1)	1 (7.1)	1 (20.0)	1 (50.0)	1 (20.0)
Walk test below cut-off	4 (26.7)	5 (35.7)	4 (28.6)	3 (60.0)	1 (50.0)	3 (60.0)
LAS below cut-off	11 (73.3)	10 (78.6)	11 (78.6)	3 (60.0)	0 (0.0)	3 (60.0)
Quality of life						
Global QoL below cut-off	9 (60.0)	6 (42.9)	9 (64.3)	5 (100.0)	2 (100.0)	3 (60.0)
Physical function below cut-off	10 (66.7)	7 (50.0)	8 (57.1)	4 (80.0)	1 (50.0)	4 (80.0)

MST, Malnutrition Screening Tool; LAS, Leisure Activity Score; QoL, Quality of Life; PG-SGA, Patient-Generated Subjective Global Assessment.

## Data Availability

The data presented in this study are available on request from the corresponding author.

## Supplementary Materials

The following supporting information can be downloaded at: https://www.mdpi.com/article/10.3390/curroncol30010054/s1, Online Supporting Material S1: Bioelectrical Impedance, Impedimed BIS, User manual; Online Supporting Material S2: JAMAR Plus+ User Manual; Online Supporting Material S3: EWGSOP2 algorithm for case-finding, making a diagnosis and quantifying severity of sarcopenia.
