# Supplementary material for: Poor Muscle Status, Dietary Protein Intake, Exercise Levels, Quality of Life and Physical Function in Women with Metastatic Breast Cancer at Chemotherapy Commencement and during Follow-Up
Source: Curr Oncol. 2023 Jan 5;30(1):688–703. doi: 10.3390/curroncol30010054 (PMC9857792; doi:10.3390/curroncol30010054)
Supplement: Supplementary file 1 [file curroncol-30-00054-s001.zip › curroncol-2055055-Supplementary Material S1.pdf]

Supplementary Material S1 - Developed Bioelectrial Impedance Impedimed BIS User Manual

<u>Populations not suitable for this measure:</u>

- **Pregnant women**
- **Patients with a pacemaker**

<u>What do you need?</u>

- Impedimed suitcase
- Pen
- Piece of paper
- Scales
- Stadiometer
- Impedimed SFB7 set (stored in second drawer of filing cabinet), charged

<u>Instructions:</u>

- Check the battery status on the display
    - Choose SETUP button to see battery

Step 1: Calibration

- Calibrate the device before use
    - One a day is sufficient

1. Remove clips from leads and connect the leads to the <u>calibration test cell</u> according to the colour-coding
2. Switch ‘on’ the machine using the small white button on the front
3. On the main screen, press the “test” button
4. Press the “start” button to make a test reading (you will see a “calculating” screen and after that, a “passed” screen signifies the device and leads are in a good condition)
5. De-attach the leads from the calibration device and re-attach the clips

Step 2: Prepare your patient

1. Measure and record height and weight
2. Record patient’s DOB, URN and age
1. Make sure the patient has emptied their bladder before the test
2. Have the patient take off their right shoe and sock (if applicable)
3. Ask patient to remove any jewellery, metal on the body or metal in clothes (e.g. belt, coins in wallet)
4. Position the patient on the bed with the **right** side of the body towards you (use left side in case the subject has an IV in the left arm)

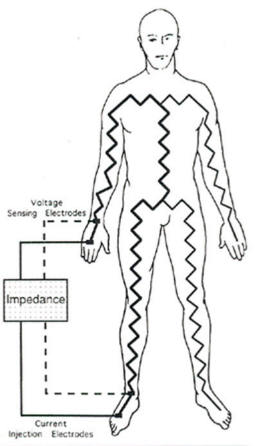

Standard placement of electrodes for whole-body impedance measurements.

5. The legs should be apart, making sure to not touch each other. If needed, place a pillow in between the legs.
6. Instruct the patient to rest for at least 5 minutes
7. Clean the skin of the wrist, hand, ankle and foot with an alcohol wipe
8. Place electrodes on the skin of the wrist, hand, ankle and foot

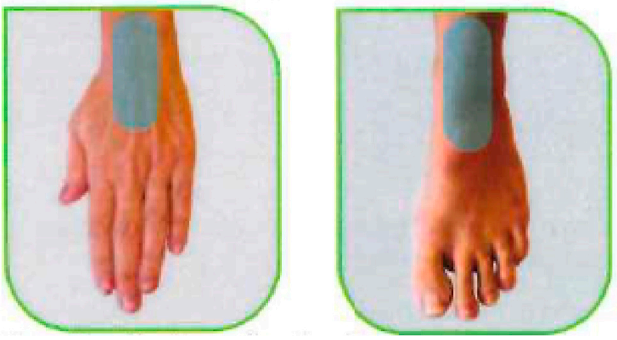

9. Make sure the electrodes are **5 cm** apart

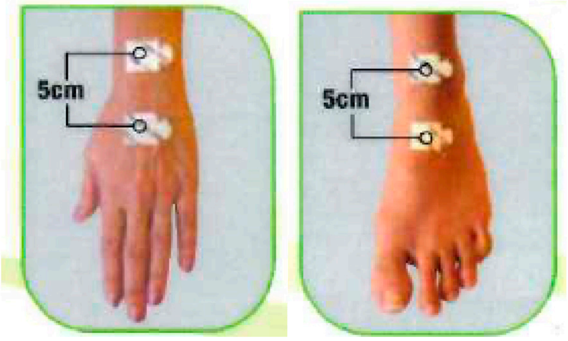

10. Attach an alligator clip to each of the probe ends of the four leads
11. Use the alligator clips to connect each lead to the tab portion of the electrode

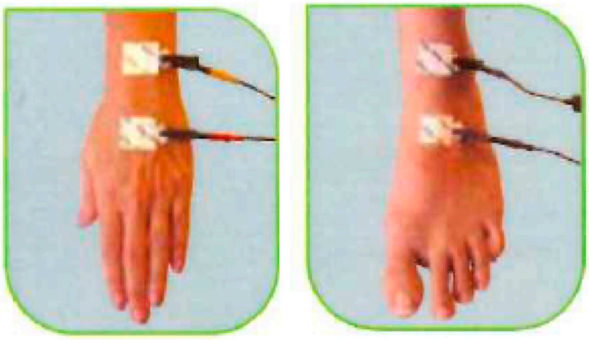

12. Apply leads according to the colour code (YELLOW = right wrist; RED = surface of right hand; BLUE = right foot on the ankle; BLACK = dorsal surface of right foot).
13. Press firmly to attach each colour coded lead plug to the similar colour locking socket on the device one by one.
14. Go back to main menu and click on MEASURE
15. Insert URN as file name and click ‘edit’ at the ‘patient details’ box
16. Enter gender, height, age and weight in this screen
17. Press OK

Step 3: Measure

1. Press MEASURE
2. Check electrode placement as suggested on screen
3. Press START
4. Record the results
5. Clean the leads of the device with a detergent wipe. *For patients under contact precautions (the dark green signs) wipe over with the disinfectant wipe twice. Allow it to dry in between.*
6. Use reference values to interpret the results

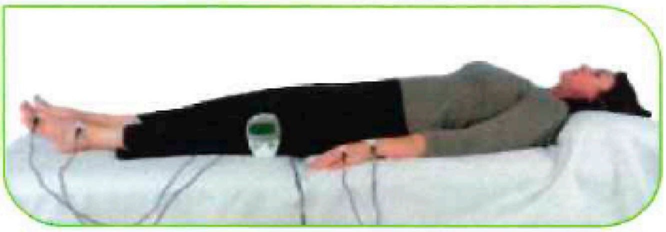

Typical set-up for measurement.

Online Supporting Material 3 - Developed JAMAR PLUS User Manual

What you need:

- JAMAR PLUS+ (Stored in 3rd drawer in filing cabinet)
- Pen
- Paper
- 2 x AAA batteries (if needing recharging); batteries are replaced on the back of the device

Instructions:

1. Check the patient's grip on the JAMAR PLUS+ before commencing: If the patient has smaller-average sized hands, adjust the grip position of the JAMAR PLUS+ to the 2nd lowest rung; if the patient has average-large sized hands, move the grip of the JAMAR PLUS+ to the 3rd lowest rung. To remove the grip position, push the lower end of the handle so that the slotted portion rotates away from the lower shaft. Allow it to then separate from the top shaft. Choose the preferred position and replace the top part of the handle onto the chosen rung, then rotate the lower part of the handle back onto the shaft until it clicks into place.

2. Use the "on/off" button to turn the JAMAR PLUS+ device on.

3. Check that the units of measurement is in kg rather than lbs. This can be seen on the right side of the display. If lbs is highlighted, remove the battery cover on the back of the device. There is a switch under the cover where you can switch lbs to kg.

4. Use the patient's left hand for the test: To select the hand and mode of the test, press the button "select test" until only the "L" is shown in the top left corner on the display.

5. The patient will perform 3 tests for an average score. To choose the number of tests the patient will complete, press the button "# of trials" until the number "3" is highlighted on the top of the display.

6. To begin the test, ask the patient to hold the JAMAR PLUS+ in their left hand while seated. Their left arm should be at a 90 degree angle.

7. Have the patient grasp the JAMAR PLUS+ gently to that the palm fits comfortably to the rear of the instrument

8. Press the "start" key and the number "1" will appear and flash at the top of the display.
9. Give the patient encouragement to squeeze the grip as hard as they can. Record the reading on the display

10. Give the patient 30 seconds recovery and press "test" to repeat for the second, and then third tests.

11. Be sure to record each reading for all 3 tests and transfer measurements to data sheet with patients de-identified number

12. Press the "reset" key to clear the previous settings before moving to the next patient to take measures.

13. When finished using the JAMAR PLUS+, use the "on/off" button toturn off.
