# Supplementary material for: Poor Muscle Status, Dietary Protein Intake, Exercise Levels, Quality of Life and Physical Function in Women with Metastatic Breast Cancer at Chemotherapy Commencement and during Follow-Up
Source: Curr Oncol. 2023 Jan 5;30(1):688–703. doi: 10.3390/curroncol30010054 (PMC9857792; doi:10.3390/curroncol30010054)
Supplement: Supplementary file 1 [file curroncol-30-00054-s001.zip › curroncol-2055055Supplementary Material S2.pdf]

Supplementary Material S2 - Developed JAMAR PLUS User Manual

What you need:

- JAMAR PLUS+ (Stored in 3rd drawer in filing cabinet)
- Pen
- Paper
- 2 x AAA batteries (if needing recharging); batteries are replaced on the back of the device

Instructions:

1. Check the patient's grip on the JAMAR PLUS+ before commencing: If the patient has smaller-average sized hands, adjust the grip position of the JAMAR PLUS+ to the 2nd lowest rung; if the patient has average-large sized hands, move the grip of the JAMAR PLUS+ to the 3rd lowest rung. To remove the grip position, push the lower end of the handle so that the slotted portion rotates away from the lower shaft. Allow it to then separate from the top shaft. Choose the preferred position and replace the top part of the handle onto the chosen rung, then rotate the lower part of the handle back onto the shaft until it clicks into place.

2. Use the "on/off" button to turn the JAMAR PLUS+ device on.

3. Check that the units of measurement is in kg rather than lbs. This can be seen on the right side of the display. If lbs is highlighted, remove the battery cover on the back of the device. There is a switch under the cover where you can switch lbs to kg.

4. Use the patient's left hand for the test: To select the hand and mode of the test, press the button "select test" until only the "L" is shown in the top left corner on the display.

5. The patient will perform 3 tests for an average score. To choose the number of tests the patient will complete, press the button "# of trials" until the number "3" is highlighted on the top of the display.

6. To begin the test, ask the patient to hold the JAMAR PLUS+ in their left hand while seated. Their left arm should be at a 90 degree angle.

7. Have the patient grasp the JAMAR PLUS+ gently to that the palm fits comfortably to the rear of the instrument

8. Press the "start" key and the number "1" will appear and flash at the top of the display.
9. Give the patient encouragement to squeeze the grip as hard as they can. Record the reading on the display

10. Give the patient 30 seconds recovery and press "test" to repeat for the second, and then third tests.

11. Be sure to record each reading for all 3 tests and transfer measurements to data sheet with patients de-identified number

12. Press the "reset" key to clear the previous settings before moving to the next patient to take measures.

13. When finished using the JAMAR PLUS+, use the "on/off" button toturn off.
