# Supplementary material for: Poor Muscle Status, Dietary Protein Intake, Exercise Levels, Quality of Life and Physical Function in Women with Metastatic Breast Cancer at Chemotherapy Commencement and during Follow-Up
Source: Curr Oncol. 2023 Jan 5;30(1):688–703. doi: 10.3390/curroncol30010054 (PMC9857792; doi:10.3390/curroncol30010054)
Supplement: Supplementary file 1 [file curroncol-30-00054-s001.zip › curroncol-2055055Supplementary Material S3.pdf]

Supplementary Material S3. EWGSOP2 algorithm for case-finding, making a diagnosis and quantifying severity of sarcopenia.

**Stage**	**Muscle Mass** [1]	**Muscle Mass** [2]		**Physical Function** [3]
Pre-sarcopenia	Low			
Sarcopenia	Low	Low	*OR*	Low
Severe Sarcopenia	Low	Low	*AND*	Low

[1] L3 skeletal muscle index ≤38.5 cm^2/m^2 (50).
[2] Handgrip strength <16kg (11).
[3] 4m walk test speed ≤0.8m/s (11).
